# Conservative treatment of idiopathic scoliosis according to FITS concept: presentation of the method and preliminary, short term radiological and clinical results based on SOSORT and SRS criteria

**DOI:** 10.1186/1748-7161-6-25

**Published:** 2011-11-28

**Authors:** Marianna Białek

**Affiliations:** 1 FITS Center, Jawor, Poland

**Keywords:** idiopathic scoliosis, FITS concept, patterns of posture, functional compensation, results of treatment, physiotherapy

## Abstract

**Background:**

Conservative scoliosis therapy according to the FITS Concept is applied as a unique treatment or in combination with corrective bracing. The aim of the study was to present author's method of diagnosis and therapy for idiopathic scoliosis FITS-Functional Individual Therapy of Scoliosis and to analyze the early results of FITS therapy in a series of consecutive patients.

**Methods:**

The analysis comprised separately: (1) single structural thoracic, thoracolumbar or lumbar curves and (2) double structural scoliosis-thoracic and thoracolumbar or lumbar curves. The Cobb angle and Risser sign were analyzed at the initial stage and at the 2.8-year follow-up. The percentage of patients improved (defined as decrease of Cobb angle of more than 5 degrees), stable (+/- 5 degrees), and progressed (increase of Cobb angle of more than 5 degrees) was calculated. The clinical assessment comprised: the Angle of Trunk Rotation (ATR) initial and follow-up value, the plumb line imbalance, the scapulae level and the distance from the apical spinous process of the primary curve to the plumb line.

**Results:**

In the Group A: (1) in single structural scoliosis 50,0% of patients improved, 46,2% were stable and 3,8% progressed, while (2) in double scoliosis 50,0% of patients improved, 30,8% were stable and 19,2% progressed. In the Group B: (1) in single scoliosis 20,0% of patients improved, 80,0% were stable, no patient progressed, while (2) in double scoliosis 28,1% of patients improved, 46,9% were stable and 25,0% progressed.

**Conclusion:**

Best results were obtained in 10-25 degrees scoliosis which is a good indication to start therapy before more structural changes within the spine establish.

## Introduction

Idiopathic scoliosis, occurring in 2-3% of the growing age population, is a developmental deformity of the spine and of the trunk. Numerous medical experts (physiotherapists, orthopaedists) have been trying to find effective ways to treat scoliosis. This is why there are so many methods and means regarding non-surgical treatment, including the use of corrective braces. However, idiopathic scoliosis by definition is a disorder of unknown origin, therefore treatment is in response to symptoms. Literature relating to conservative treatment results in children with idiopathic scoliosis comprises many publications concerning the effects of treatment by means of exercises combined with the use of rigid braces [1-5], or the use of soft braces [6-8], rigid versus flexible spinal orthosis [9] or with rigid braces only [10]. In 2011, an updated systematic review on physical exercises in the treatment of adolescent idiopathic scoliosis was published, indicating the level 1b according to the Oxford Centre for Evidence-based Medicine being the evidence level for the current publications concerning results of physiotherapy treatment [11].

In author's clinical practice, we receive children with various spinal deformities, including defects of posture, mild scoliosis (Cobb 10-25°), moderate curvatures (Cobb 26-40°) requiring physiotherapy and corrective bracing, and severe scoliosis (Cobb angle greater than 50°), who underwent surgical treatment or who for various reasons are not operated (refusal, contra-indication).

The method Functional Individual Therapy of Scoliosis (FITS method) has been created for idiopathic scoliosis. However, beneficial influence of this method could be predicted for other structural and in non structural spinal deformities.

In the literature the percentage of braced patients who progressed significantly and had to undergo surgery in the case of 26-40° scoliosis, oscillates between 8% and 41% [12,13].

Effective scoliosis treatment involves radiological and clinical improvement decreasing all the trunk, pectoral girdle, pelvic girdle and lower limb asymmetries. Currently, certain instruments presented in the published literature comprise a methodological reference framework Scoliosis Research Society (SRS) criteria [14] and clinical reference framework Society of Scoliosis Orthopedic and Rehabilitation Treatment (SOSORT) criteria [15]. Following those criteria, it is postulated to be possible to compare the results of different treatments. The results of brace treatment have been described in Nachemson's and Janicki's publications [10,16].

There are also several reports which include the results of treatment of scoliosis of 10-25° by means of exercises only [17-24].

This article describes FITS method, which was created and developed by the two authors: Marianna Białek PT, PhD and Andrzej M'hango PT, MSc in 2004 for diagnosis and therapy of structural and not structural scoliosis. The author of this publication-Marianna Białek, reports her preliminary, short term results of scoliosis treatment with FITS method, with the average observation period of 2.08 years. The methodology is based on the SRS and SOSORT criteria. Although a retrospective cohort study with comparison with natural history, it is the first one to present the preliminary results after 2,08 years of treatment with FITS method.

### Description of the FITS method

Main principles of FITS concept:

1. To make the child aware of existing deformation of the spine and the trunk as well as indicate a direction of scoliosis correction.

2. To release myofascial structures which limit three-plane corrective movement.

3. To increase thoracic kyphosis through myofacial release and joint mobilization.

4. To teach correct foot loading to improve position of pelvis and to realign scoliosis.

5. To strengthen pelvis floor muscles and short rotator muscles of the spine in order to improve stability in the lower trunk.

6. To teach the correct shift of the spine in frontal plane in order to correct the primary curve while stabilizing (or maintaining in correction) the secondary curve.

7. To facilitate of three-plane corrective breathing in functional positions (breathing with concavities).

8. To indicate correct patterns of scoliosis correction and any secondary trunk deformation related to curvature (asymmetry of head position, asymmetry of shoulders' lines, waist triangles and pelvis).

9. To teach balance exercises and improvement of neuro-muscular coordination with scoliosis correction.

10. To teach correct pelvis weight bearing in sitting and correction of other spine segments in gait and ADL.

### Main Body

FITS concept consists of three main stages:

#### Stage I

Examination of child with scoliosis using classical assessment but also in terms of FITS method.

#### Stage II

Preparation for correction-examination, detection and elimination of myofascial restriction which limits three-plane corrective movement by using different techniques of myofascial relaxation.

#### Stage III

Three-dimensional correction-building and fixation of new corrective patterns in functional positions.

#### Stage I. Patient examination and making the child aware of the trunk deformity

**Figure 1:**
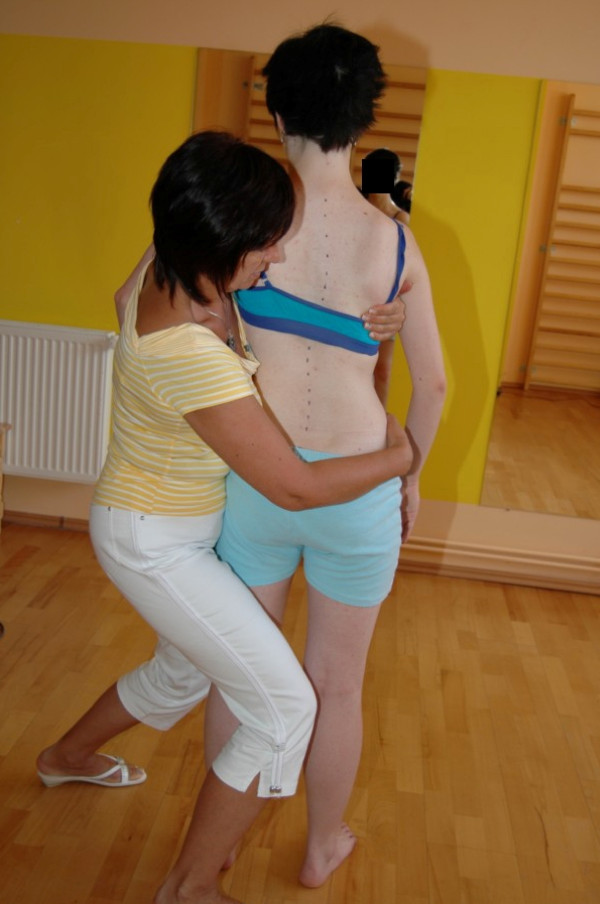
**Examination of flexibility of the scoliotic spine in functional positions**.

Classical assessment includes: history, course of treatment, X-ray analysis and examination of patient in three different planes. Afterwards clinical assessment is performed according to FITS:

□ Distance from plumb line to: anal cleft, the apex of primary and secondary curve, the edge of the scapula,

□ checking position of both scapulas,

□ observation of type and location of compensation,

□ position of pelvis and measurement of angle trunk rotation (ATR) using Bunnell scoliometer,

□ assessment of the settings of the lower limbs in standing and gait,

□ assessment of the length of muscles in lower limbs, pelvic girdle, shoulder girdle and trunk,

□ Assessment of possibilities for scoliosis correction in standing and sitting (Figure 1).

**Figure 2:**
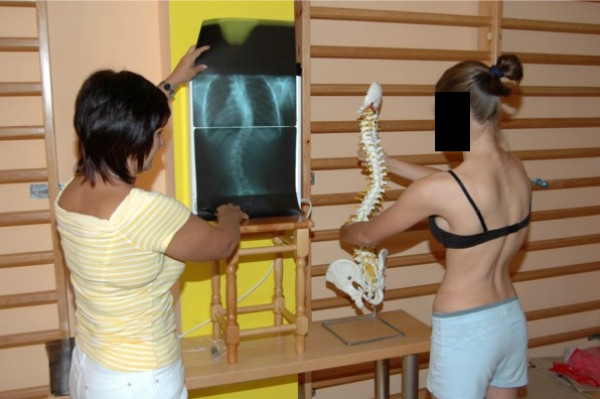
**Making child aware of trunk deformity due to scoliosis**.

The authors of this concept emphasize a great role of child awareness of the type of scoliosis but also trunk deformation caused by scoliosis. We analyze the X-ray with the child, the three-dimensional position of scoliosis on the model of the spine and we show direction of correction (Figure 2). In author's opinion making the child a partner not a subject of therapy, increases significantly motivation to exercise and improves the effects of therapy at the same time.

Long lasting experience in study with scoliosis has shown significant myofascial limitations in the area of many muscle chains [25]. The limitations are particularly visible while attempting to perform passive corrective movement, in functional positions. By making corrective movement, the therapist is able to feel which myofascial structures should be treated first. In order to indicate direction of therapy and effectiveness of therapeutic procedures we test corrective movement during each session. Corrective movement at the beginning of therapy can be done only in one plane-shift, rotation or flexion/extension. In further stages of therapy three-dimensional corrective movement should be included.

#### Stage II. Preparation for correction

After patient is completely examined we move on to relaxation of structures restricting correction by using techniques like: contract-relax technique, passive and active myofascial release [26,27], trigger points [28], joint mobilization [29,30] and neuromobilisation [31]. These techniques are often used in the area of myofascial bands according to Myers:

□ SBL (superficial back line)

□ DFL (deep front line)

□ LL (lateral muscle line)

□ SL (spiral muscle line)

□ SFL (superficial front line)

**Figure 3:**
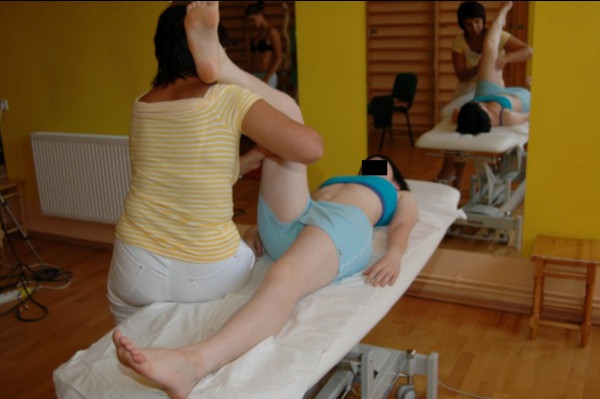
**Active myofascial relaxation for hamstrings and erector spinae**.

**Figure 4:**
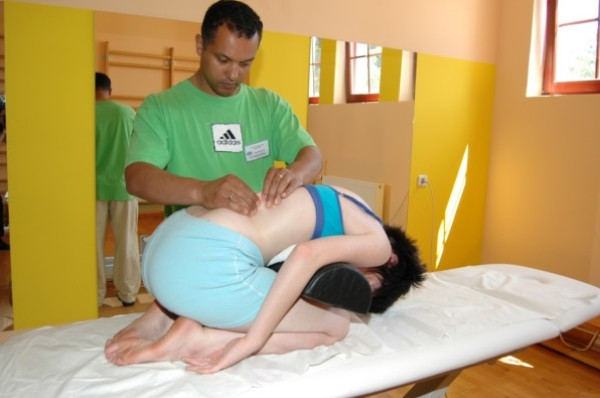
**Active myofascial relaxation for erector spinae**.

One of the first symptoms of scoliosis is deformation of the physiological shape of the spine in sagittal plane (particularly flattening of thoracic kyphosis) [32]. Therefore therapy should include normalisation of tension between both muscle band responsible for spine alignment in sagittal plane which is: SBL (superficial back line) (Figure 3, 4) and DFL (deep front line).

Correction in the frontal plane is preceded by relaxation of contracted or very tight muscles which is a part of LL (lateral line). An important role is played here by tensor fasciae latae muscle and tractus iliotibialis, that are very shortened on the side of convexity of Th/L scoliosis. Muscle energy technique should be performed in prone position on the edge of the bench (lower extremity on the concave side of scoliosis rests on the floor). The therapist should correct Th/L scoliosis by decreasing of deeper waist triangle and derotation of scoliosis. After this correction we can notice the real shortening of these structures (at this moment lower extremity on convex side moves laterally). To the group of LL muscles we have to also add lateral part of abdominal oblique muscles external and internal.

**Figure 5:**
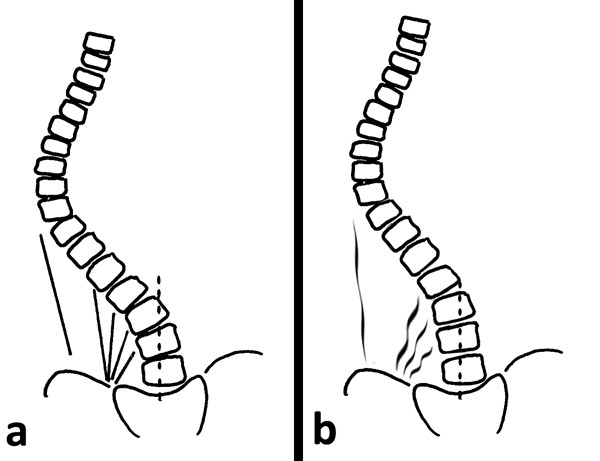
**The myofascial structures limiting the corrective shift (a-before therapy, b-after therapy)**.

In addition, the muscles between the apex of the curve and iliac crest make corrective lateral shift more difficult (lower part of latissimus dorsi and erector spine, posterior part of internal abdominal oblique muscle, posterior layer of quadrates lumborum and lateral part Th/L fascia) (Figure 5). Their pelvis attachments on the convex side of Th/L scoliosis should be released in functional position-sitting and standing. This will facilitate the corrective shift.

Particularly attention should be paid to relaxation of the quadratus lumborum muscle in the Th/L scoliosis. Posterior layer of fibers should be subject to relaxation on the convex side (in the lower part-between the iliac crest and processes transverses of the lumbar spine), while the frontal layer of this fibers on the concave side of scoliosis (in the upper part-between the lumbar vertebrae and rib XII.

Derotation of the scoliotic spine is the most difficult corrective movement for therapist as well as for child. There are two muscle groups responsible for this movement: SBL (short paraspinal rotators) and SL (muscles of spiral line). At first we should release muscles of SL, because they are more superficial. The crucial area of our therapy is the Anterior Superior Iliac Spine (ASIS) area where we observe diversity of mechanical tension going out from it. The force vectors of muscles work in different direction so precision is important in myofascial release in order to obtain desired therapeutic effect. This is followed by release of short spinal rotators-first in prone position, then in sitting (during this maneuver the patient actively performs scoliosis derotation). Derotational movement of the trunk is particularly difficult in double major scoliosis where two opposite directions of vertebral body rotation are located. This is the reason why the relaxation of these muscles for Th segment should be performed by patient's shoulder movement, for Th/L segment by pelvis movement. The secondary curve of scoliosis should always be stabilized.

**Figure 6:**
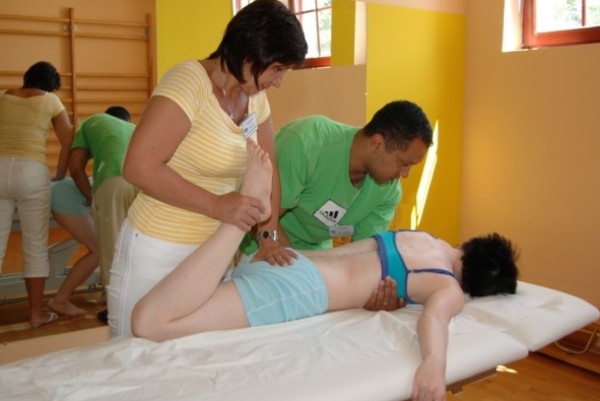
**Active relaxation for rectus femoris with scoliosis derotation maneuver**.

Significant therapeutic effects can be obtained by relaxation of deep frontal line. It is responsible for the profile of the spine in the sagittal plane but also has an impact on derotation of scoliosis by myofascial connection with the anterior longitudinal ligament, which changes its placement depending on Cobb angle magnitude and direction of corpus of vertebrae rotation. The patient's posture in correction when releasing muscles of deep front line can have an impact on clinical change around costal hump (Figure 6).

Pelvis floor muscles (which is the part of DFL) improve stabilization of lower trunk which results in correction of upper curve of scoliosis. Obviously before DFL release, myofascial tension in SFL should be decreased, because SFL keeps the tension in deeper tissues.

After removing myofascial restrictions which make corrective movement difficult, improving flexibility and mobility of scoliosis, we go on to the next stage of therapy.

#### Stage III. Three-plane correction

**Figure 7:**
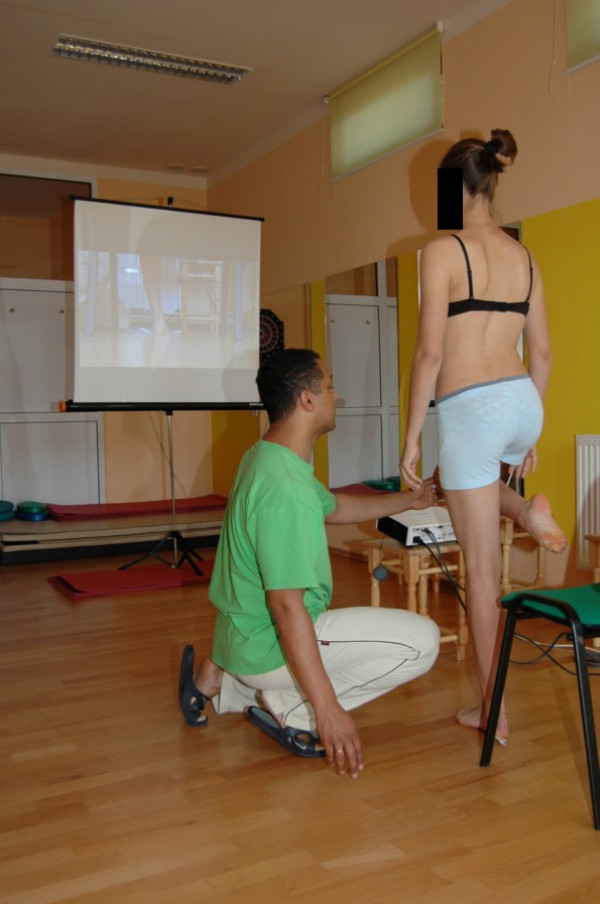
**Sensory-motor control training on one leg**.

**Figure 8:**
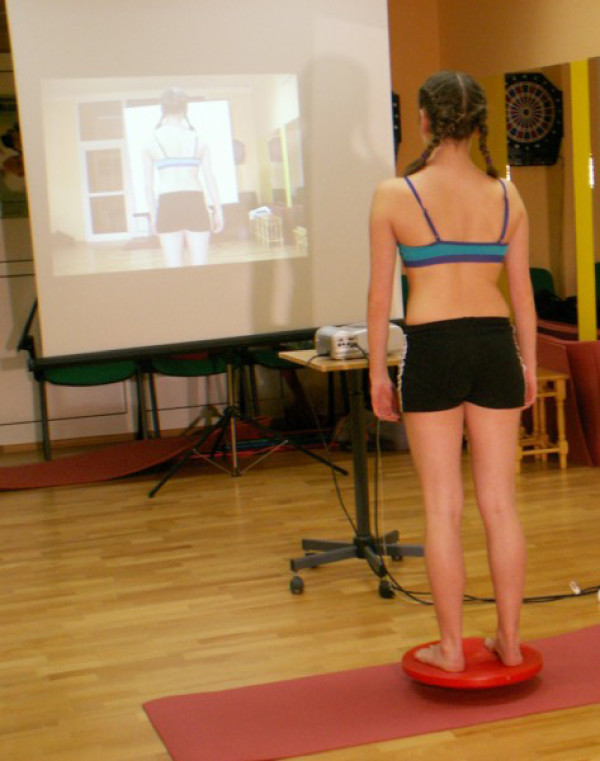
**Sensory-motor control training on the balance trainer**.

To build and stabilize new corrective patterns of posture in functional positions we start from correct foot loading using sensory motor balance training according to Greenman [33] (Figure 7, 8).

We begin from sitting position moving to standing, reducing gradually support surface and making exercise more difficult (wobble boards, sensory cushions). We also use biofeedback which significantly improves therapeutic effects [34].

**Figure 9:**
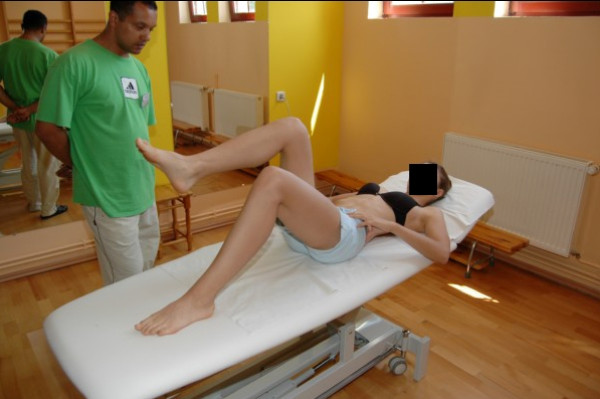
**Stabilization of lower trunk with 3-dimensional correction of scoliosis**.

**Figure 10:**
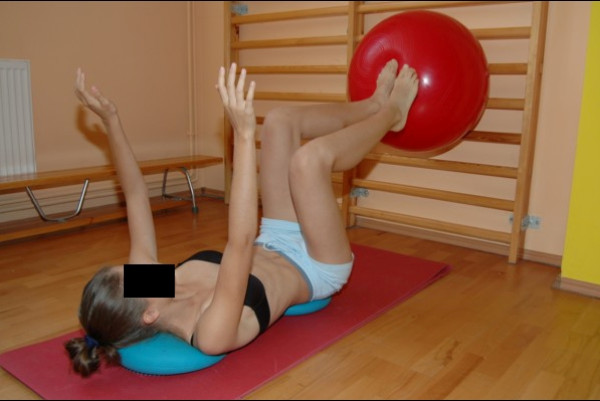
**Stabilization of lower trunk with pillows sensorimotor and the ball**.

Observing children with scoliosis we notice unsettled stabilization of the lower part of trunk, especially during everyday activities [35-37] (Figure 9, 10). On clinical examination we observe enlarged lumbar lordosis. However, after lateral X-ray analysis, the increased lordosis comprise lower lumbar spine while flattening of lordosis is observed in the upper lumbar spine. Stabilization exercise of the lower part of trunk is essential for the study of corrective patterns of the upper part of trunk and shoulder girdle.

In obtaining corrective movement children have particular difficulty doing lateral shift correction in the frontal plane, mainly due to increased tension of the myofascial tract between the iliac crest and the apex of scoliosis. It is recommended to exercise shift in functional positions (sitting and standing). The technique of combination of isotonic contractions is indicated. In case of double major scoliosis we need to be aware that the shift of the primary curve should be done with stabilization (or correction) of secondary curve.

**Figure 11:**
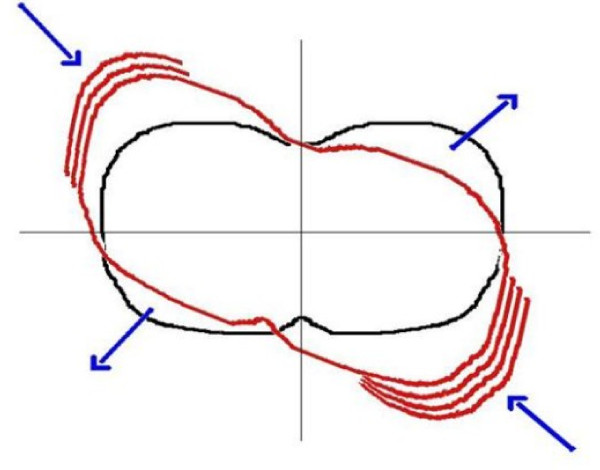
**Corrective chest movement for thoracic scoliosis**.

Facilitation to three-plane corrective breathing should be done after diaphragm release and restoring the best possible joint mobility in thoracic spine and thorax-derotation breathing exercise (Figure 11). This technique of breathing has been used by Schroth since 1921 and it has been described in many papers [38,39]. The effectiveness of the mentioned exercise can be improved by adding elongation of scoliosis concavity by using upper and lower limb patterns. In every case attention should be paid to correct position in sagittal plane. The exercise is an essential element of costal hump correction particularly when performed in functional positions.

Teaching patterns which correct scoliosis and all other trunk deformations (associated with curvature) is done in open and closed kinematic chain exercise, with use of Thera Band [40]. Each limb pattern consists of correction in sagittal, frontal and transversal plane. The choice of each element of every corrective pattern depends on Cobb angle, size and direction of trunk rotation, position of the spine in sagittal plane and location of functional compensation [41-44].

**Example: **for left single curve scoliosis with left rotation, without compensation, with decreased thoracic kyphosis, increased lumbar lordosis-we suggest following patterns:

Pattern of right upper extremity (PruE)- Flexion, Adduction/Abduction and internal rotation (Flx, Add/Abd, int rot).

Pattern of left upper extremity (PluE)-Extension, Adduction/Abduction and external rotation (Ext, Add/Abd, ext.rot).

Pattern of right lower extremity (PrlE)- Extension, Adduction/Abduction and external rotation (Ext, Add/Abd, ext.rot).

Pattern of left lower extremity (PllE)- Flexion, Adduction/Abduction and internal rotation (Flx, Add/Abd, int rot).

These patterns are to be held until the moment when on the concave side of the primary curve (above and below this curve) the minor functional compensation will appear-less than 3-4 degree of rotation [45]. This compensation concerns only soft tissues, not structures seen on

X-rays. At this moment changing of patterns for upper extremities should be done in the direction to elongate both sides. The rotation of right upper extremity should be changed in neutral rotation. In lower extremities patterns we only change the rotation component on the side of existing functional compensation (we give neutral rotation).

Pattern of right upper extremity (PruE)- Flexion, Adduction/Abduction and neutral rotation-a position between external rotation and internal rotation (Flx, Add/Abd, neut. rot).

Pattern of left upper extremity (PluE)-Flexion, Adduction/Abduction and external rotation (Flx, Add/Abd, ext.rot).

Pattern of right lower extremity (PrlE)- Extension, Adduction/Abduction and neutral rotation-a position between external rotation and internal rotation (Ext, Add/Abd, neut.rot).

Pattern of left lower extremity (PllE)- Flexion, Adduction/Abduction rotation (Flx, Add/Abd, int rot).

**Figure 12:**
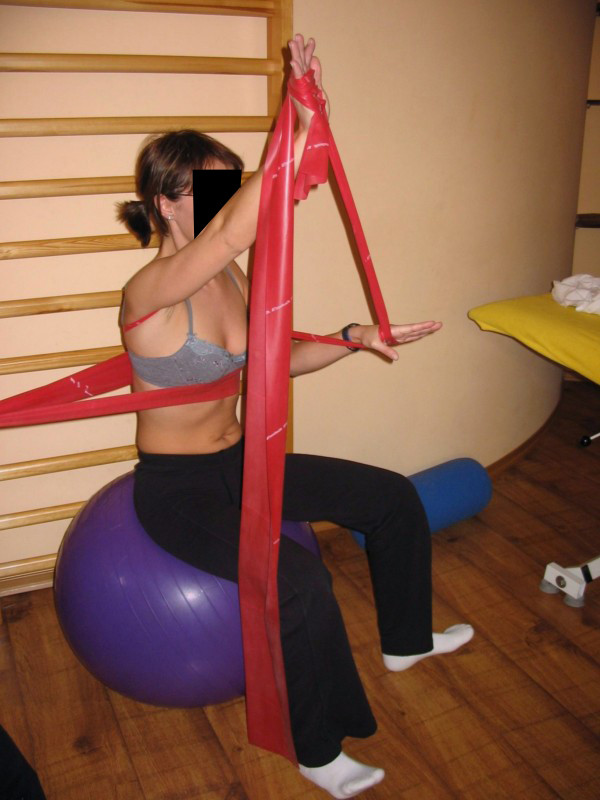
**An example of corrective pattern in functional position**.

**Figure 13:**
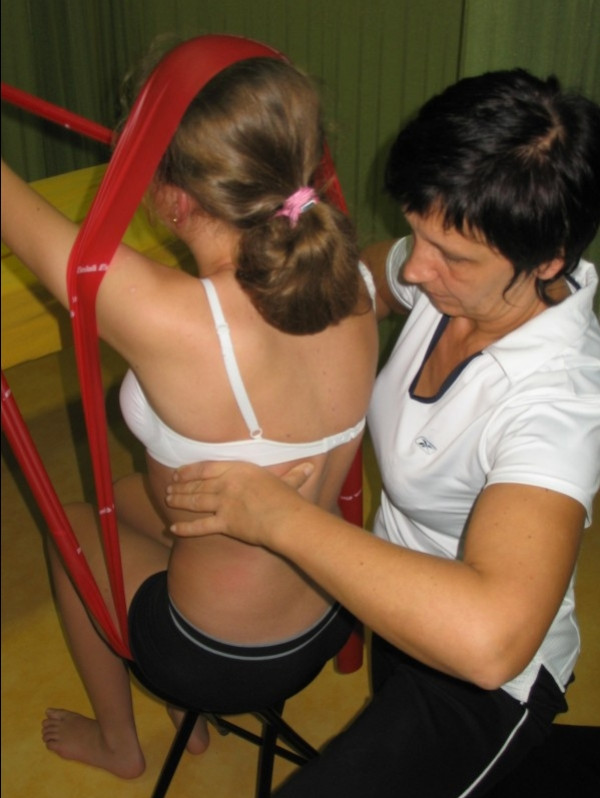
**An example of corrective pattern in functional position**.

**Figure 14:**
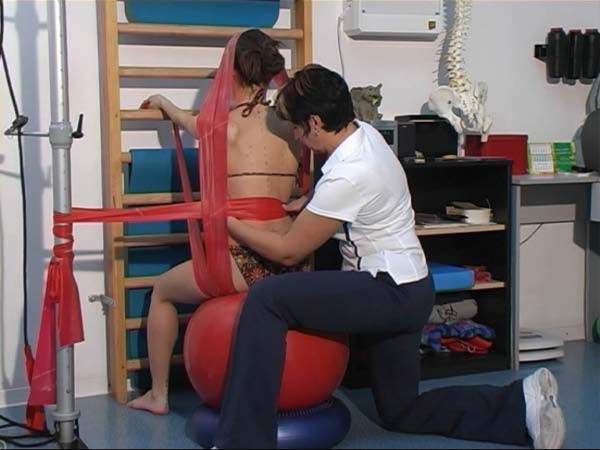
**An example of corrective pattern in functional position**.

This modification of patterns will cause elongation movement of the upper trunk and further building of compensation in lumbar spine in frontal and sagittal plane. The rotation component on the side of primary scoliosis will be sustained, but changed to neutral on the right. The choice of presented patterns will cause decrease of primary scoliosis without structural compensation (additional compensation curve seen on X-ray). For each child patterns should be chosen individually and modified in the appropriate time according to clinical changes (Figure 12, 13, 14).

In scoliosis treatment, the most desirable outcome is correction of the primary structural curve in three planes. However in many cases it might be too difficult due to considerable degree of curvature or insufficient correctivity of curvature. In those cases we believe that creating and developing compensatory (functional) curves (in soft tissues only) above and below the primary curve is beneficial. Correction of two or three structural curves is harder and less effective than only one structural curve. Scoliosis of small Cobb's angle range are more possible to correct. By creating functional compensation we can achieve balanced posture and good clinical effect. It is very important to be able to establish at which level the compensation is the most desirable and more amenable to correction (the most desirable when, where, and if to decrease or add the compensation). The type and size of functional compensation can be evaluated by measuring trunk rotation using Bunnell's scoliometer [45].

FITS is a complex, asymmetrical and individual therapy that can be used at any age and Cobb angle. It requires the child to take an active part in the process of therapy guided by an experienced therapist. FITS therapy is conducted in an in-patient clinic and on an out-patient basis (2-week course of treatment). It is recommended to work in an interdisciplinary group of specialists including: physiotherapist, orthopedist and psychologist.

Considering trunk deformity due to scoliosis the authors of this study suggest an individually adjusted program of exercises. There are two different types of goals: general (what means decreasing of scoliosis angle, stabilization of scoliosis angle, preparing for surgical intervention, clinical improvement after surgery) and specific (correction of shoulders, scapulas and head position, pelvis derotation, facilitation of three-plane corrective breathing, facilitation of corrected posture pattern, improvement of lumbo-pelvic stabilization.

### Material

Out of 374 children who received treatment according to the FITS concept between 2005 and 2010, those patients who at the moment of the beginning of the treatment were 10 years or more older, presented Cobb from 10 do 40 degrees and Risser sign 0 or 1 or 2, were retrospectively analyzed. Total number of the children included in the study amounted to 115. They were analyzed in two groups separately:

**Group A**-68 girls and 10 boys, who received the FITS therapy without bracing. The group comprised children that age was > or = 10 years, with the Cobb angle between 10° and 25° and the Risser sign between 0 and 2. The children were classified into two subgroups:

□ subgroup A1-single thoracic (Th) or thoracolumbar (Th/L) or lumbar (L) curve (52 children)

□ subgroup A2-double scoliosis: thoracic (Th) and thoracolumbar (Th/L) or lumbar (L) curves (26 children).

**Group B**-34 girls and 3 boys who received the FITS therapy combined with bracing. The group comprised children that age was > or = 10 years, with the Cobb angle between 26° and 40° and the Risser sign between 0 and 2. The children were classified into two subgroups:

□ subgroup B1-single thoracic (Th) or thoracolumbar (Th/L) or lumbar (L), (5 children)

□ subgroup B2-double scoliosis: thoracic (Th) and thoracolumbar (Th/L) or lumbar (L) curves (32 children).

All patients used only FITS therapy. Apart from performing exercises according to the FITS concept, the children from the B group, due to the high Cobb angle, were instructed to wear the Chêneau brace for 13 to 21 hours a day. At follow-up examination the patients were asked to reveal the actual time of brace wearing.

The number of female/male patients, the average age, average Cobb angle, the number of girls after menarche as well as the number of patients with Risser sign value of 0, 1 or 2 are given in Table 1. In case of double curve scoliosis both the thoracic and the thoracolumbar/lumbar Cobb angle were taken for calculations.

**Table 1:** Initial characteristics of the patients.

Group	Subgroup	Sex F/M	Mean age (years)	Mean Cobb angle (degrees)	Girls after menarche	Risser 0/1/2
A	A1	44/8	11.9	17.7	18	31/2/19
	
10°-25°	A2	24/2	11, 1	Th 16.5	5	9/2/5
						
				ThL/L 15.9		

B	B1	3/2	12.8	28.6	1	3/0/2
	
26°-40°	B2	31/1	12.9	Th 31.0	16	18/3/11
						
				ThL/L 31.2		

In the clinical examination, the following parameters were measured: (1) angle of trunk rotation (ATR) at the Th and ThL/L or L-level before and after treatment, measured with the Bunnell scoliometer and expressed in degrees, (2) distance between the plumb line (projected from the middle of the external occipital protuberance) and the gluteal sulcus, measured with a ruler and expressed in centimeters, (3) distance between the tip of the spinous process of the apex of primary scoliosis and the plumb line, expressed in centimeters, and (4) the scapulae level asymmetry, measured with the Bunnell scoliometer and expressed in degrees (Table 2).

**Table 2:** Clinical parameters at study entry.

Group	Subgroup	Angle of trunk rotation (ATR), Bunnell degrees		Distance: plumbline-gluteal sulcus (cm)		Distance: plumb line-apex of primary scoliosis (cm)		Scapulae level asymmetry (Bunnell degrees)	
		
		average	range	average	range	average	range	average	range
A	A1	4, 7	0-13	1.3	0-2, 5	0.9	0, 3-2, 5	3.1	1-6
	
10°-25°	A2	Th 5.5	0-11	1.1	0-2, 5	1, 0	0, 5-1, 8	3.3	1-9
								
		ThL/L 4.0	0-12						

B	B1	6, 4	6-14	0.5	0-1, 5	1.2	1-1, 5	6.4	3-8
	
26°-40°	B2	Th 7.9	2-19	1.4	0-3, 4	1.5	0, 2-2, 5	4.6	0-11
								
		ThL/L 6.7	1-14						

## Method

### Course of FITS treatment

Children received individual treatment according to the FITS concept once a month (60 minutes). This therapy was performed by the author herself. Between the individual therapy meetings, at home, once a day (45 minutes), the patients performed adequately selected and prescribed set of exercises. In cases when the physiotherapists educated in FITS were accessible, the patients received individual treatment at their places of residence. The patients were educated to sit in a correct position during classes at school and at homework. Twice a year, a two-week in-patient physiotherapy was offered in the form of a winter or summer rehabilitation camp. Forty-four children out of 78 participated in at least one two-week rehabilitation camp.

### Methodology of evaluation

For children who were wearing the brace, a questionnaire was used to reveal the number of hours the brace is being worn.

Clinical studies were performed in all patients by the author of the manuscript.

At follow-up (after FITS therapy) the clinical parameters were re-assessed and the patients were subjected to X-ray analysis for the Cobb angle. The patients had the X-rays taken in the place where they lived. Cobb angle was measured by the treating physician.

The percentage of children in whom the Cobb angle decreased by more than 5°, percentage of children in whom the Cobb angle was stable during the observation period (± 5°) and percentage of children in whom the Cobb angle increased by more than 5° were calculated at follow-up.

### Statistical analysis

Kolmogorov-Smirnov test was used to check the normality. Paired t test was applied to compare the values before and after therapy in case of normal distribution. Wilcoxon matched pairs test was used if the normal distribution was not present.

## Results

The mean observation period was 2.08 years (range 1-5 years). Twenty-six patients finished the treatment while 89 are still under treatment.

No patient from the 115 FITS treated patients underwent scoliosis surgery. Out of all the patients treated according to the FITS concept between 2005 and 2010 (374 children), 7 girls underwent surgery. They were not included in this study since at the beginning of the therapy, they did not fulfil the SRS criteria because the Cobb angle was over 45°.

### In group A (78 patients-scoliosis of 10-25°)

**Figure 15:**
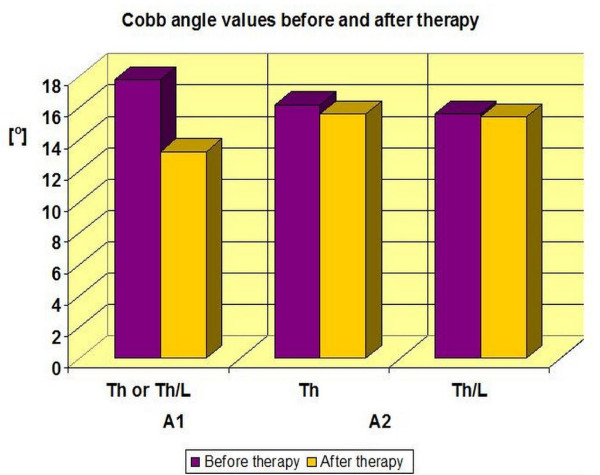
**Cobb angle values before and after therapy in A group**.

**Figure 16:**
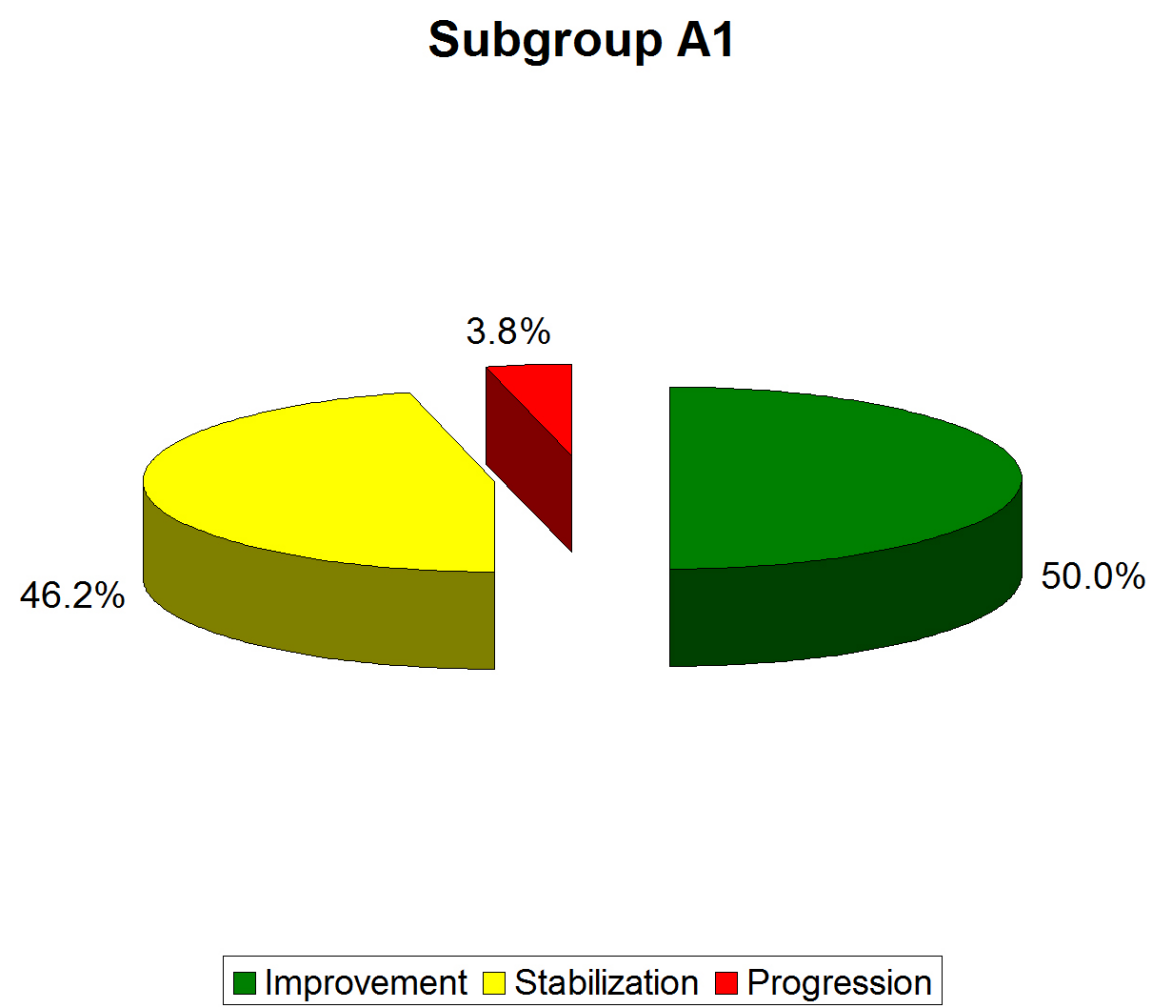
**Percentage of improvement, stabilization and progression in A1 subgroup**.

- A1 (single curve scoliosis)-the average Cobb angle decreased from 17.7° (± 4.2°) to 13.0° (± 5.9°). In this subgroup, 50.0% of the patients improved by more than 5 degrees, 46, 2% stabilized their scoliosis and 3.8% experienced progression of Cobb angle by more than 5° (Table 3, 4, Figure 15, 16). The results were statistically significant p < 0.0001.

**Table 3:** Cobb angle values (in degrees) in A group-exercises only (paired t test).

Subgroup	N	Region	Before therapy			After therapy			P
				
			Mean	± SD	Range	Mean	± SD	Range	
A1	52	Th or ThL	17.7	± 4.2	10-24	13.0	± 5.9	4-28	**< 0.0001**

A2	26	Th	16.5	± 5.9	10-25	15.6	± 9.2	0-40	0.52
	
	26	Th/L or L	15.9	± 7.3	10-25	15.4	± 8.9	0-29	0.72

**Table 4:** Numbers and percentage values of scoliosis improvement, stabilization and progression

Subgroup	Improvement		Stabilization		Progression	
	
	Number	Percentage	Number	Percentage	Number	Percentage
A1	26	50.0%	24	46, 2%	2	3.8%

A2	13	50.0%	8	30.8%	5	19.2%

B1	1	20.0%	4	80.0%	0	0.0%

B2	9	28.1%	15	46.9%	8	25.0%

**Figure 17:**
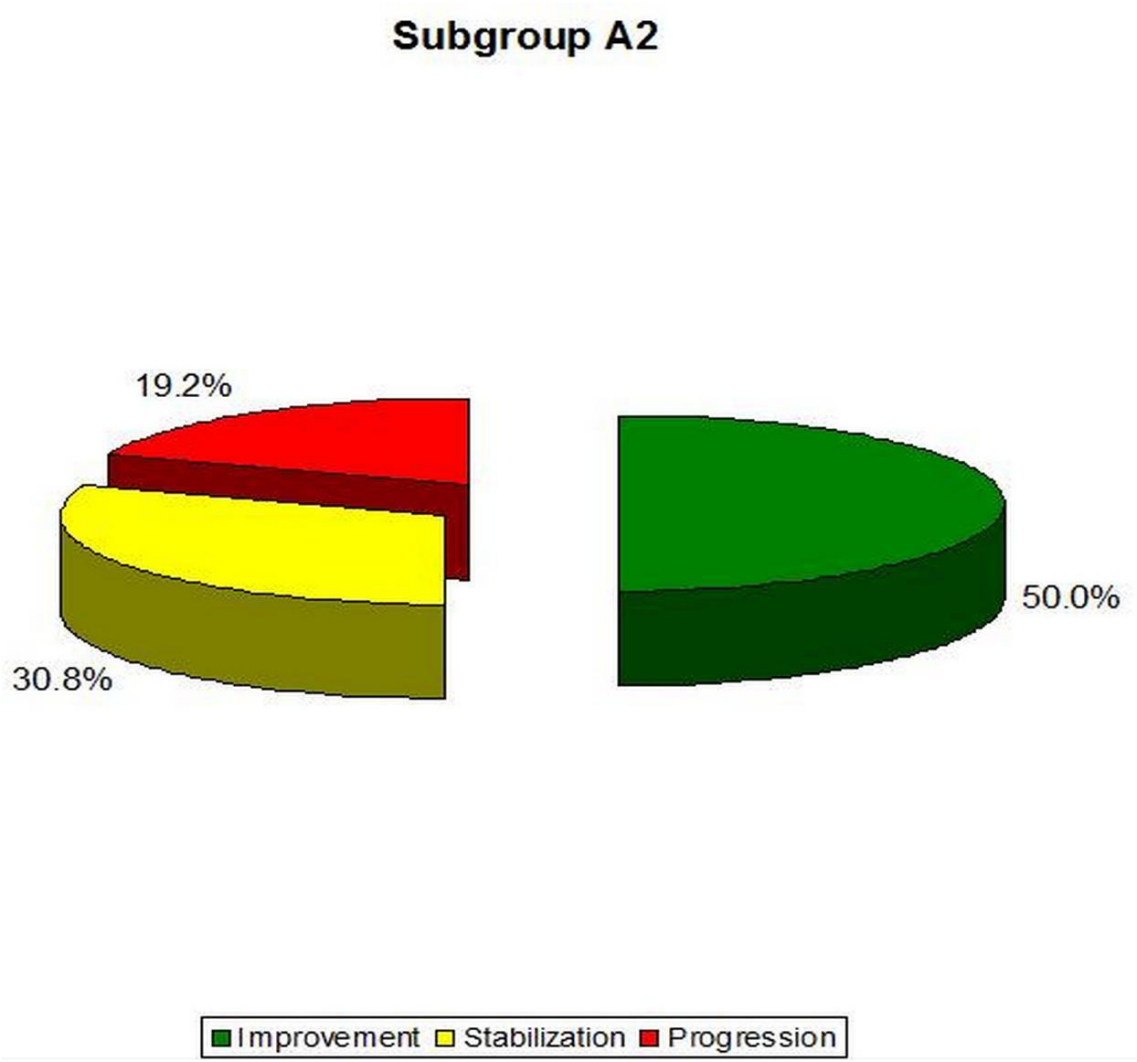
**Percentage of improvement, stabilization and progression in A2 subgroup**.

- A2 (double curve scoliosis)-the average Cobb angle in the Th region decreased from 16.5° (± 5.9°) to 15.6° (± 9.2°), and in the Th/L or L region from 15.9° (± 7.3°) to 15.4° (± 8.9°). In this subgroup, 50.0% of patients improved by more than 5 degrees, 30.8% obtained scoliosis stabilization and 19.2% obtained Cobb angle progression of more than 5° (Table 3, 4, Figure 15, 17). The results obtained in this group were not statistically significant (p = 0, 52 for Th and p = 0, 72 for the Th/L).

Group B (37 patients-scoliosis of 26-40°)

Based on the answers of the patients and the parent it was found that the actual time of bracing amounted to 12-14 hours a day (time ordered by the physician amounted to 13-21 hours a day).

**Figure 18:**
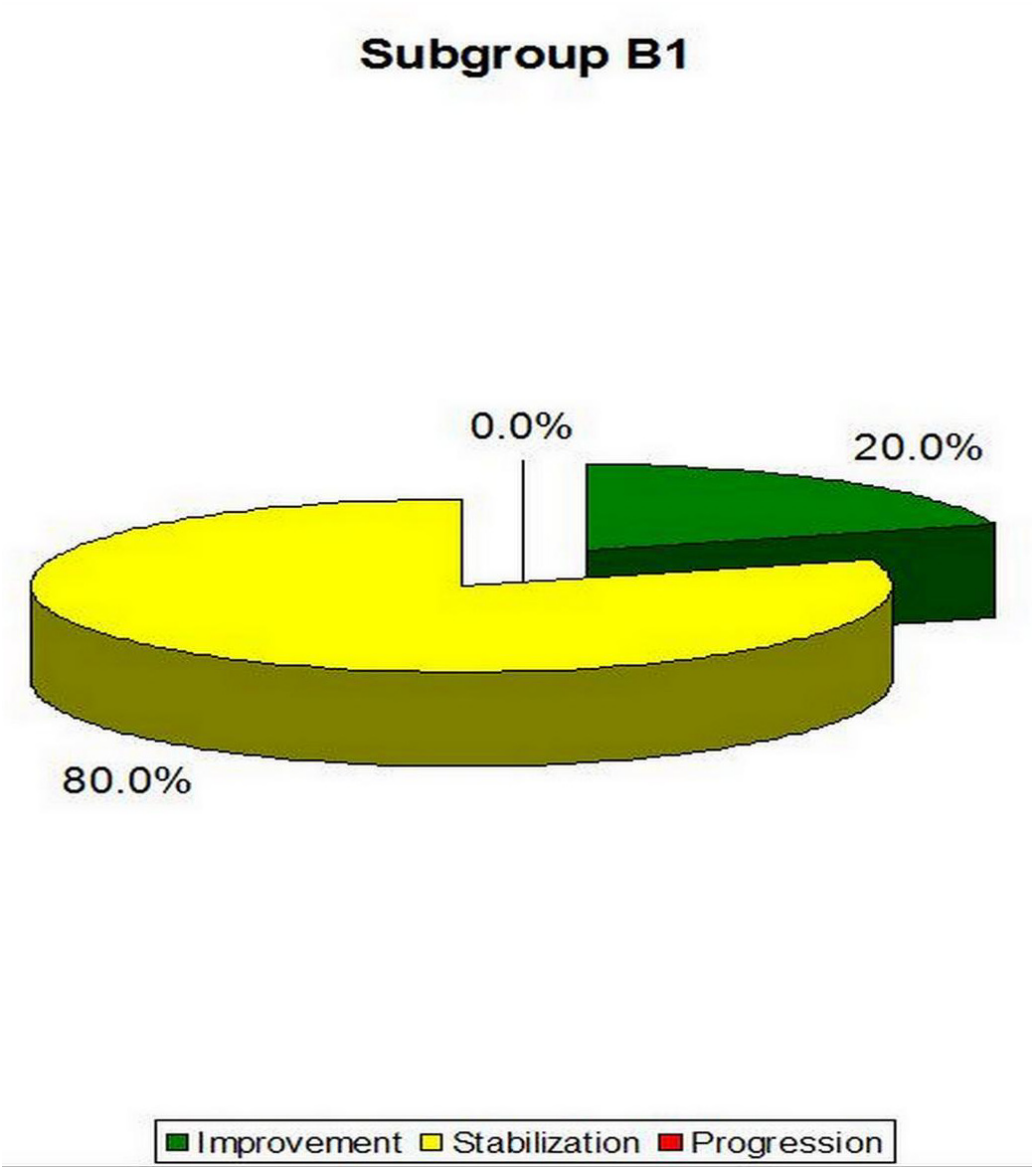
**Percentage of improvement, stabilization and progression in B1 subgroup**.

- B1 (single curve scoliosis)-the average Cobb angle decreased from 28.6° (± 4.2°) to 26.4° (± 6.8°). It constitutes 20.0% of the patients who obtained improvements of more than 5 degrees, 80.0% of the patients who obtained scoliosis stabilization and 0% of the patients with Cobb angle progression of more than 5° (Table 5, Figure 18.). The results obtained in this group were not statistically significant (p = 0, 41) possibly due to small number of patients in the group.

**Table 5:** Cobb angle values (in degrees) in B group-exercises and part time bracing (paired t test).

Subgroup	N	Region of scoliosis	Before therapy			After therapy			P
				
			Mean	± SD	Range	Mean	± SD	Range	
B1	5	Th or ThL	28.6	± 4.2	26-35	26.4	± 6.8	16-34	0.41

B2	32	Th	31.0	± 6.1	20-40	31.9	± 6.9	18-48	0.48
	
	32	Th/L	31.2	± 5.0	20-40	30.0	± 6.8	18-45	0.22

**Figure 19:**
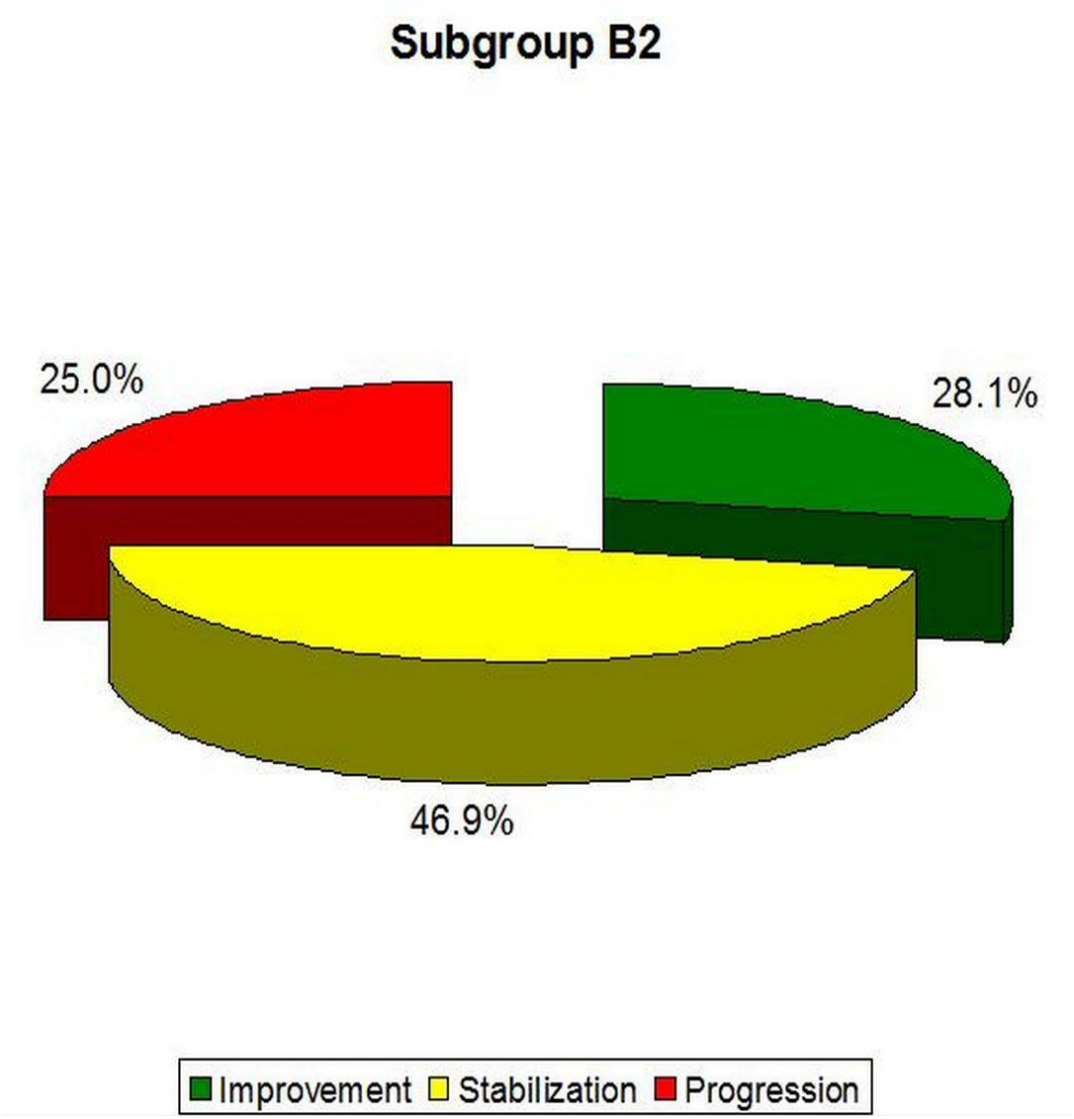
**Percentage of improvement, stabilization and progression in B2 subgroup**.

- B2 (double curve scoliosis)-the average Cobb angle in Th region increased from 31.0° (± 6.1°) to 31.9 (± 6.9°), while it decreased within the Th/L or L region from 31.2° (± 5.0°) to 30.0° (± 6.8°). In this group, 28.1% of patients obtained improvement of more than 5 degrees, 46.9% obtained stabilization of scoliosis, and 25.0% obtained Cobb angle progression of more than 5° (Table 5, Figure 19). The results obtained in this group also were not statistically significant (p = 0, 48 for Th and p = 0, 22 for Th/L scoliosis).

Progression of scoliosis more than 5° was the highest in the thoracic scoliosis in group B2 (18.7%), slightly less-in Th and Th/L or L component of double scoliosis in group A2 (15.4%). Progression in both curves was greater in group A2 (11.5%) than in group B2 (3.1%) (Table 6).

**Table 6:** Percentage of progression of more than 5° divided into Th and Th/L or L curve.

Subgroup	N patients in each group	N patents with progression	% progression in either curve	N patients with progression in Th curve	% progression in Th curve	N patients with progression in Th/L curve	% progression in TH/L curve	N patients with progression on both curve	% progression in both curves
A1	52	2	3, 8%	0	0%	2	3, 8%	-	-

A2	26	5	19, 2%	4	15, 4%	4	15, 4%	3	11, 5%

B1	5	0	0	0	0%	0	0%	-	-

B2	32	8	25%	6	18.7%	3	9, 4%	1	3, 1%

### Clinical evaluation of the trunk

In many children, who underwent the therapy, significant clinical changes were observed within the trunk. In particular, a statistically significant improvement was achieved in the reduction of ATR in group A of scoliosis in the single scoliosis Th/l and in double scoliosis in Th (p < 0.0001).

A similar result was obtained in group B2 in Th/L (Table 7).

**Table 7:** Values of the angle (in degrees) of trunk rotation (ATR). Significance tested with paired t test.

Subgroup	N	Level of ATR measure	Before therapy		After therapy		P
				
			Mean ± SD	Range	Mean ± SD	Range	
A1	52	Th	3.3 ± 2.9	0-12	2.7 ± 2.1	0-9	**0.0006**
	
	52	ThL/L	6.1 ± 3.4	0-13	3.9 ± 2.9	0-10	**< 0.0001**

A2	26	Th	5.5 ± 2.4	0-11	3.8 ± 2.5	0-10	**< 0.0001**
	
	26	ThL/L	4.0 ± 3.1	0-12	3.4 ± 2.5	0-9	0.21

B1	5	Th	6.4 ± 4.6	2-14	4.8 ± 5.0	0-12	0.09
	
	5	ThL/L	6.4 ± 5.5	0-10	5.2 ± 5.1	0-9	0.41

B2	32	Th	7.9 ± 4.4	2-19	6.2 ± 3.7	0-15	**0.0013**
	
	32	ThL/L	6.7 ± 3.7	1-14	4.6 ± 3.0	0-11	**< 0.0001**

Significance tested with paired t test.

Statistically significant differences in the clinical study were obtained in all groups (except group B1) to reduce the following parameters: the distance from the vertical occiput line to the gluteal sulcus, scapular asymmetry and vertical deviation of the apex for primary scoliosis (p < 0.0001). In group B1, these results have not improved significantly (Tables 7, 8, 9, 10).

**Table 8:** Values of the distance (in centimeters) from the vertical occiput line to the gluteal sulcus. Significance tested with Wilcoxon matched pairs test.

Subgroup	N	Before therapy		After therapy		P
			
		Mean ± SD	Range	Mean ± SD	Range	
A1	52	1.3 ± 1.4	0-2, 5	0.1 ± 0.2	0-0, 8	**< 0.0001**

A2	26	1.1 ± 0.6	0-2, 5	0.2 ± 0.2	0-0, 6	**< 0.0001**

B1	5	0.5 ± 0.6	0-1, 5	0.1 ± 0.2	0-0, 2	0.25

B2	32	1.4 ± 0.8	0-3, 4	0.7 ± 0.6	0-2, 5	**< 0.0001**

Significance tested with Wilcoxon matched pairs test.

**Table 9:** Values of the scapular asymmetry (Bunnell degrees). Significance tested with Wilcoxon matched pairs test.

Subgroup	N	Before therapy		After therapy		P
			
		Mean ± SD	Range	Mean ± SD	Range	
A1	52	3.1 ± 1.0	1-6	0.6 ± 1.2	0-8	**< 0.0001**

A2	26	3.3 ± 1.6	1-9	0.6 ± 1.5	0-7	**< 0.0001**

B1	5	6.4 ± 2.1	7-8	2.7 ± 3.2	0-7	0.0625

B2	32	4.6 ± 2.4	1-11	1.6 ± 1.9	0-6	**< 0.0001**

Significance tested with Wilcoxon matched pairs test.

**Table 10:** Average values (in centimeters) of vertical deviation of the apex for primary scoliosis. Significance tested with paired t test.

Subgroup	N	Before therapy		After therapy		P
			
		Mean ± SD	Range	Mean ± SD	Range	
A1	52	0.9 ± 0.4	0, 3-2, 5	0.3 ± 0.3	0-1, 5	**< 0.0001**

A2	26	1.0 ± 0.4	0, 5-1, 8	0.4 ± 0.4	0-1, 3	**< 0.0001**

B1	5	1.2 ± 0.2	1-1, 5	0.9 ± 0.7	0, 5-0, 8	0.52

B2	32	1.5 ± 0.6	0, 2-2, 5	0.9 ± 0.6	0-2, 5	**< 0.0001**

Significance tested with paired t test.

In Group A1 only two people have increased Cobb angle, but none of them did not exceed an angle of 30°.

In group A2-two out of five people received the progression of scoliosis angle, reaching over 30° or 35°. None of them reached the operating value. However, because of age, amenorrhea, and high values of Cobb angle, they are still at risk of surgery.

In group B2-only one in eight has progressed to > 48 degrees, but because skeletal maturity was reached, surgery was not required. Three people from this group during the observation period were Risser 0, so are at risk of surgical intervention (Table 11).

**Table 11:** Presentation case by case of children who progressed more than 5 degrees.

Subgroup	Age	Menarche	Before therapy					After therapy					Time observation in years
			
			Cobb angle in Th	Cobb angle in Th/L or L	ATR in Th	ATR in Th/L or L	Risser	Cobb angle in Th	Cobb angle in Th/L or L	ATR in Th	ATR in Th/L or L	Risser	
A1	11	_	0	10	0	5	0	0	23	0	1	0	1
	
	13	X	0	22	1	12	2	0	28	5	5	4	2

A2	10	_	30	28	11	4	0	**40**	29	10	4	0	1
	
	11	_	16	12	7	0	0	18	24	6	1	1	1
	
	11	_	10	10	0	7	0	27	29	1	4	0	2
	
	12	_	11	11	6	3	0	23	23	7	6	3	2
	
	10	_	21	22	6	0	0	**31**	29	8	3	3	2

B2	13	X	39	34	13	0	0	**48**	42	11	5	4	3
	
	13	_	28	23	1	0	0	**42**	27	12	1	3	2
	
	13	X	20	30	7	1	1	**32**	**33**	8	3	5	5
	
	13	_	32	38	6	14	0	**40**	**38**	9	11	1	1
	
	13	X	27	31	5	2	0	28	**38**	2	2	0	1
	
	13	_	23	33	7	10	0	**38**	**38**	6	5	0	3
	
	13	X	38	30	15	3	2	**32**	**45**	12	2	3	2
	
	12	_	20	40	8	10	0	**40**	**43**	6	8	0	1

Operating value: 48 The value seen in bold exceed 30 degrees.

### Examples of clinical and radiological improved idiopathic scoliosis

**Figure 20:**
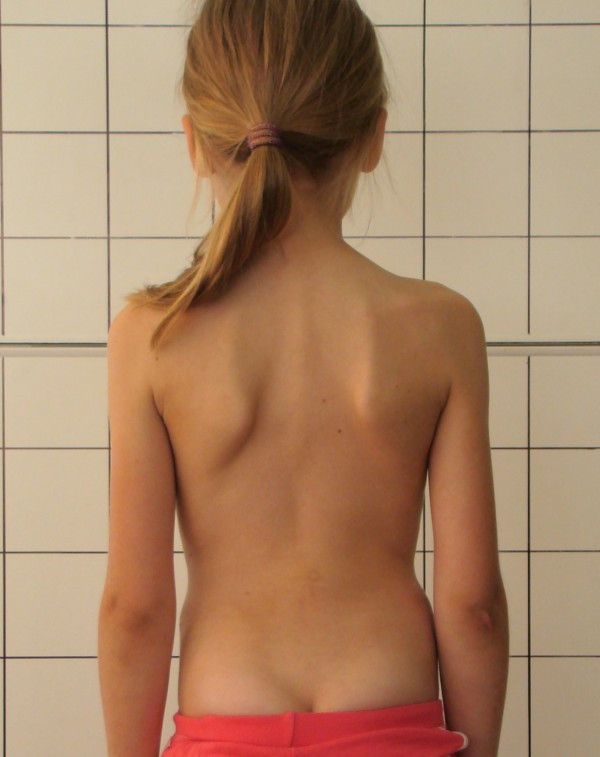
**Clinical image before therapy**.

**Figure 21:**
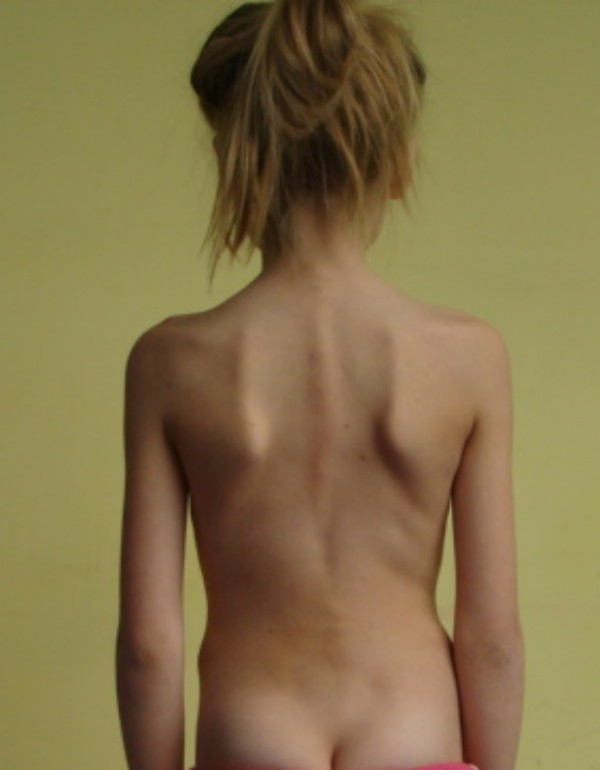
**Clinical image after therapy**.

**Figure 22:**
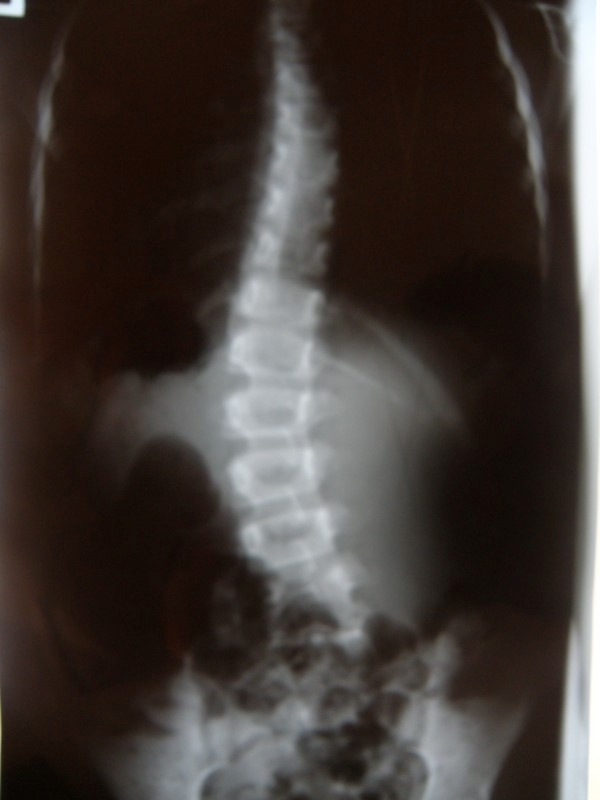
**Radiography before treatment**.

**Figure 23:**
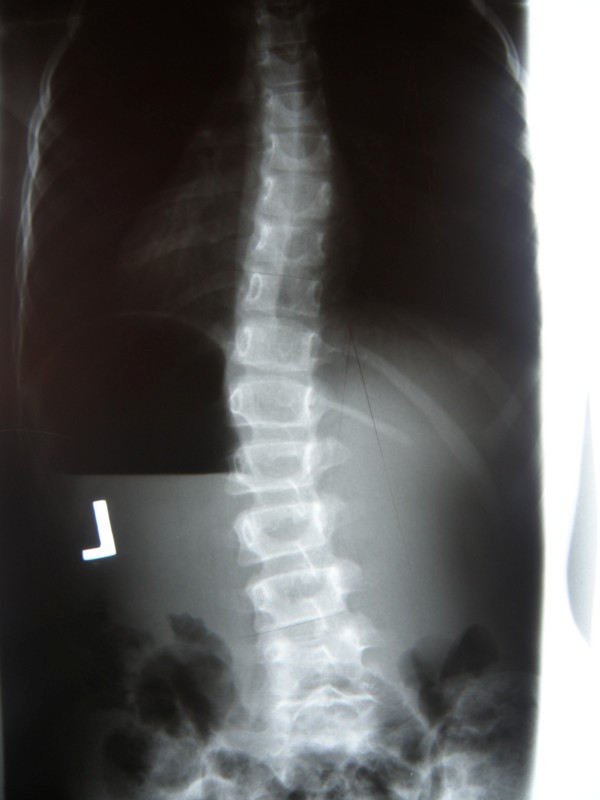
**Radiography after treatment**.

**Figure 24:**
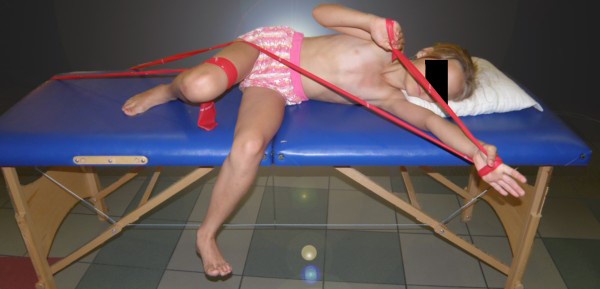
**Exercise in pattern correction**.

**Example I-**clinical and X-ray effect of improved scoliosis in A1 group (Figure 20, 21, 22, 23, 24).

Marta 10 years:

Th_5_-Th_10 _dex 31°, Th_10_-L_3 _sin 25°, Risser 0 (01.2010)

Th_5_-Th_10 _dex 17°, Th_10_-L_3 _sin 25°, Risser 0 (01.2011).

**Figure 25:**
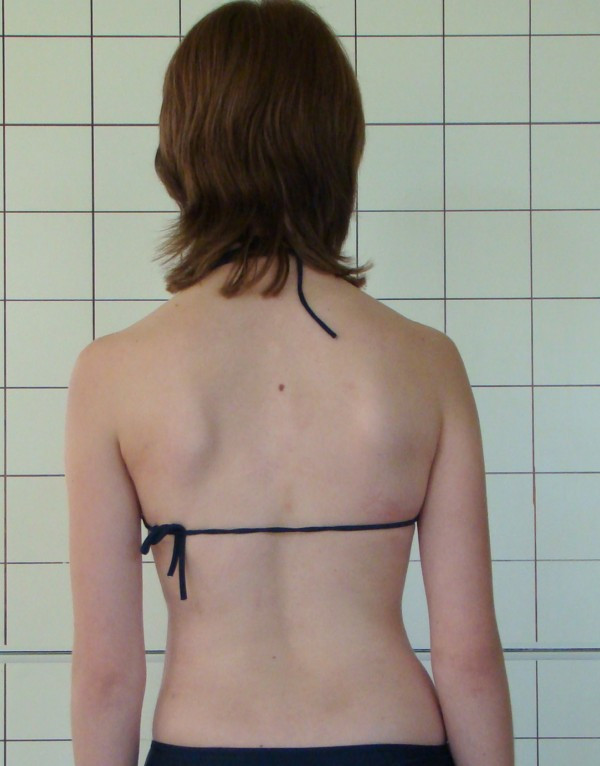
**Clinical image before therapy**.

**Figure 26:**
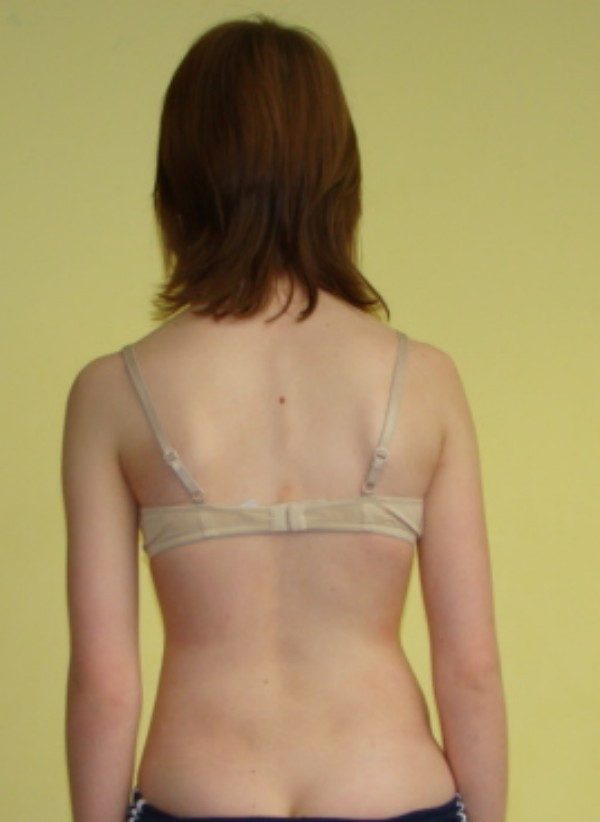
**Clinical image after therapy**.

**Figure 27:**
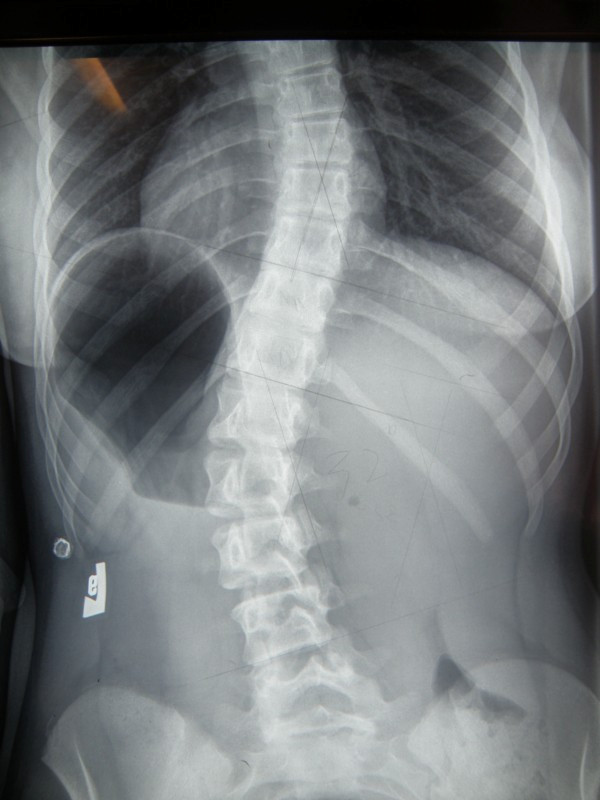
**Radiography before treatment**.

**Figure 28:**
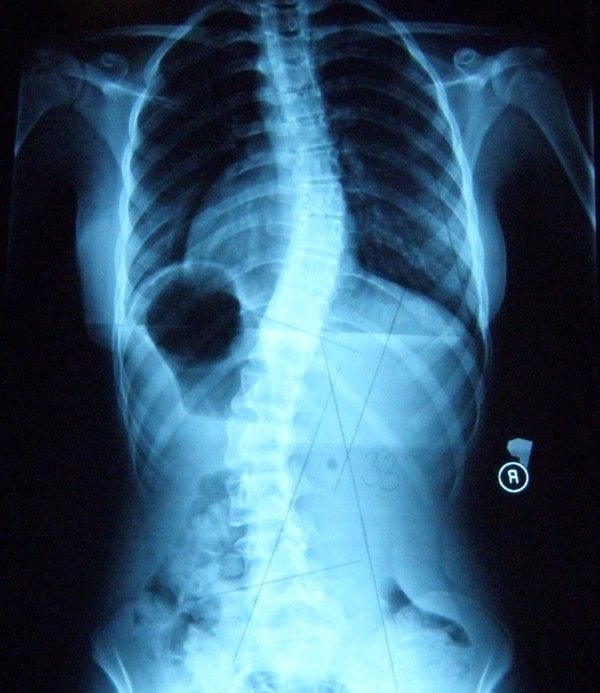
**Radiography after treatment**.

**Figure 29:**
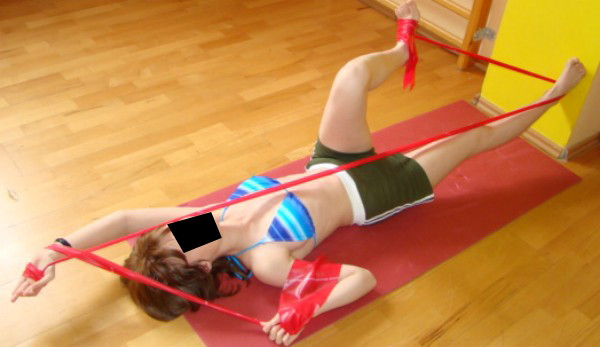
**Exercise in pattern correction**.

**Example II**- clinical and X-ray effect of stable scoliosis and classified as B2 group-scoliosis braced (Figure 25, 26, 27, 28, 29).

Barbara-12 years:

Th_5_-Th_11 _dex 29°, Th_11_-L_4 _sin 31°, Risser 2 (05.2010).

Th_5_-Th_11 _dex 31°, Th_11_-L_4 _sin 28°, Risser 3 (02.2011).

**Figure 30:**
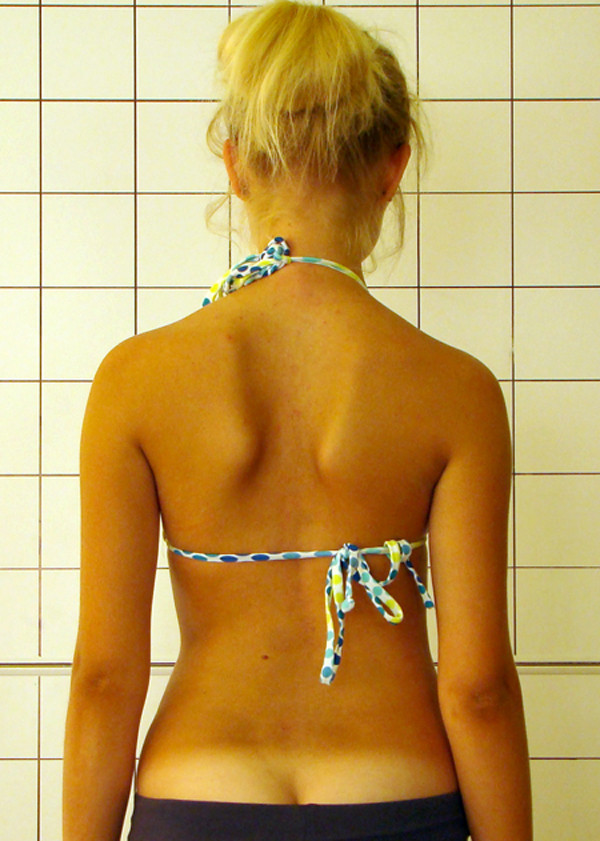
**Clinical image before therapy**.

**Figure 31:**
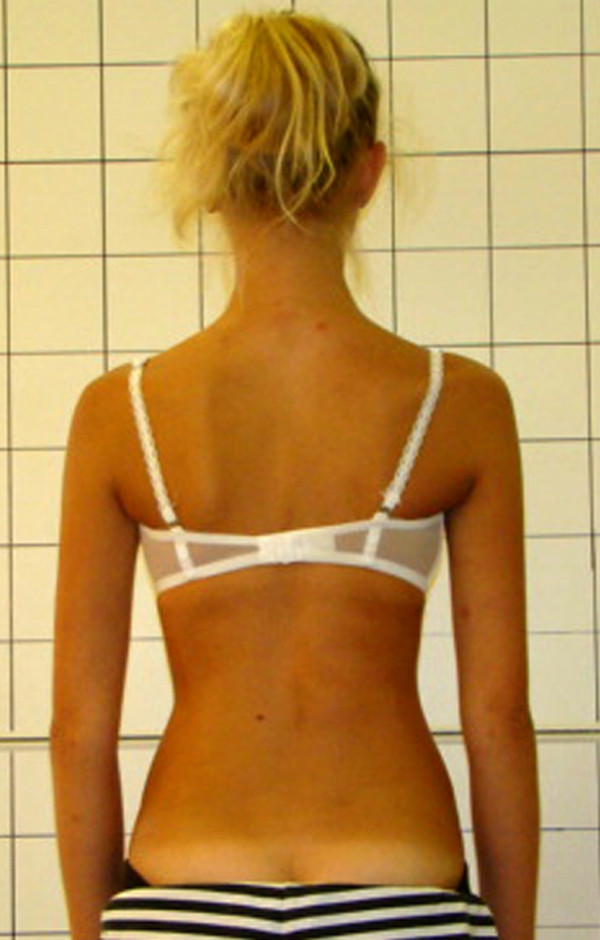
**Clinical image after therapy**.

**Figure 32:**
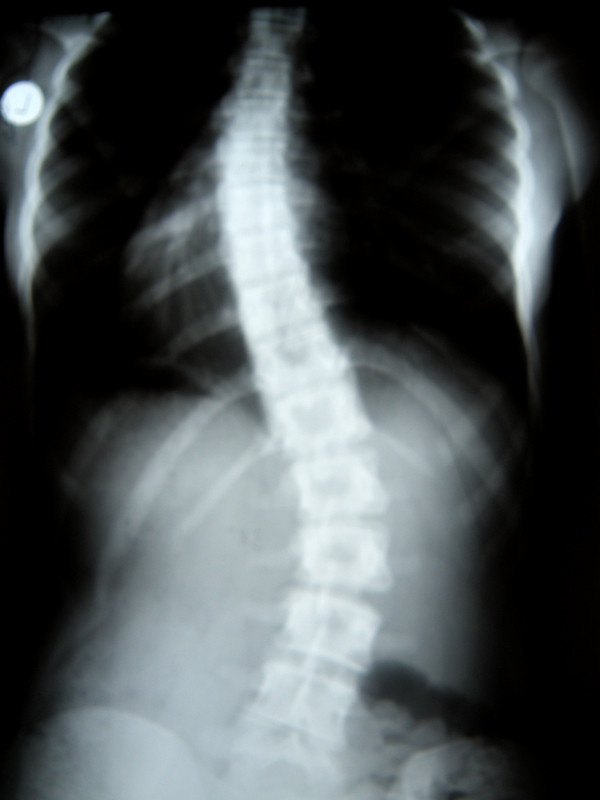
**Radiography before treatment**.

**Figure 33:**
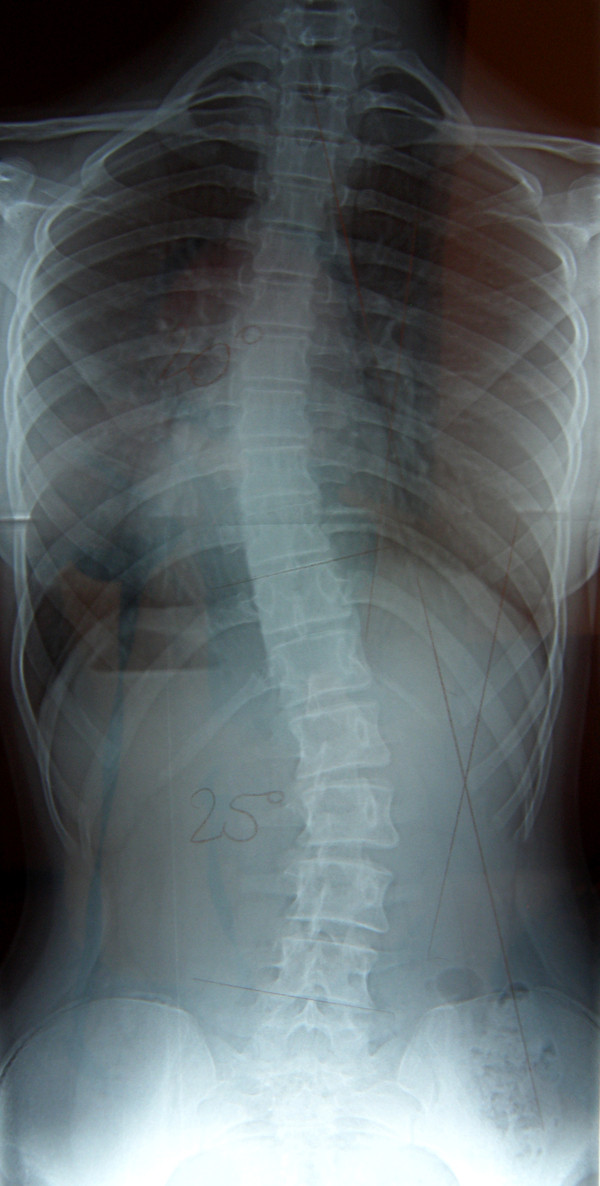
**Radiography after treatment**.

**Figure 34:**
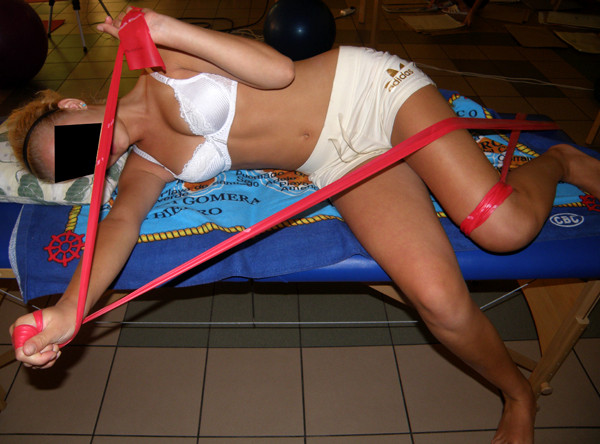
**Exercise in pattern correction**.

**Example III**- Clinical effect of corrected scoliosis in B2 group -not braced (Figure 30, 31, 32, 33, 34).

Marcelina-14 years:

Th_4_-Th_10 _sin 33°, Th_11_-L_4 _dex 38°, Risser 0 (07.2009).

Th_4_-Th_10 _sin 20°, Th_11_-L_4 _dex 25°, Risser 3 (10.2010).

## Discussion

Scoliosis is a symptom that develops in response to numerous causes including injury, infection and genetic disorders [46-48]. In most cases, treatment addresses symptoms of scoliosis is still unknown, it forces us to plan treatment of symptoms of scoliosis (any asymmetry of the trunk, incorrect loading of the lower limbs and pelvis asymmetric adjustment) rather than its cause. Improving the proper loading of the lower limbs and pelvis by proper balance setting gives a good basis for uniform loads discs and cartilage growth maturing of the spine, which is so vulnerable to asymmetric loads. Based on the study of molecular and biomechanical basis of deformation of the spine can be concluded that in most cases (except for congenital deformities of bones), soft tissue balance will be crucial to the stabilization of the curvature and/or improvement of clinical symptoms [49,50]. FITS method meets these requirements with a view to relaxation of soft tissue to reach as far as possible balanced tension. After removing soft tissue restrictions the patient can perform the correction patterns and then stabilize.

The results of treatment that combines exercises and the use of braces presented in English publications differ significantly and depend on the method of exercises and type of the braces used (soft or rigid). Some of the authors claim that using rigid braces produces good results-Nachemson et al [10]. Others point to the advantage of rigid braces over the soft ones-Wong et al [9], and vice versa-Coillard et al [6-8].

Comparing the obtained results in the examined non-braced groups of patients subjected to FITS therapy (A1 single curves-3, 8% progression, A2 double curves-15, 4% progression in thoracic curve, 15, 4% progression in thoracolumbar or lumbar curve, 11, 5% progression in both curves and 19, 2% progression in either curve) with a natural history of non-treated cases of scoliosis presented by Lonstein and Carlson [51] (single curves-17, 6% progression, double curves-27, 0% progression: 25% progression in thoracic component and 43% progression in lumbar component) and Hitesh N Modi (32% improvement and 26% progression) [52] -it can be concluded that FITS method is an effective therapy for children suffering from idiopathic scoliosis.

It is difficult to compare this manuscript data to literature data, because only few papers present detailed data in respect to curve type (single versus double), and curve size according to the SRS criteria. In A1 group (single curve 10-25°) 51% of improvement versus 3, 8% of progression was noted; comparable literature data were not found. In A2 group (double curves 10-25°) 50% of improvement and 19.2% of progression was found. Others authors treating the same curve type reported respectively: the very good results were achieved by Otman in 2005-100% improvement (from the average angle of curvature 26, 1° to 17, 8°) [17]. Mollon in 1986 described a study on one hundred and sixty children with AIS who were treated with therapy according to Lyon method. Improvement in 63% of patients was observed and could result from a small Cobb angle at the beginning of therapy (average 16°). The authors obtained better results than in the control group comprising 50 people [18]. Similarly good results presented Klisic -58% improvement and 37% progression [19] and Duconge-58% improvement and 42% progression [20]. Good result were obtained by Rigo 44, 2% improvement and 11, 6% progression [21], Durmala-in thoracic scoliosis 31% improvement and 39% progression, in lumbar scoliosis 35% improvement and 39% progression [22] and Negrini 29% improvement and 3% progression [23,24].

In the study group A2 (double scoliosis), one person demonstrated progression in the thoracic curvature still maintaining Risser 0 within 1 year of therapy. However, considering very good clinical effect, the patient refused the brace and accepted it only after further progression.

In braced patients the results were better in single curves (20% improvement, 0% progression) than in double curves. Possible causes of poor results in double scoliosis could be: technical problems in brace with space for the push and counter-push, stiffening properties of the brace, more severe and rigid primary scoliosis, insufficient daily use of brace, weak proprioception or improper approach of the child and its parents to exercising. The result obtained in the group B1 could possibly be influenced by a small number of people in this group. Being necessary to increase the sample in the future.

For each child and her/his parents it is very important if during the conservative treatment the scoliosis improves both radiologically and clinically. Quite often, radiological and clinical outcome differ significantly. Clinical improvement itself motivates children to exercise and work on their health. In author's observation, clinical improvement occurs long before the radiological improvement can be observed. In some cases author noted that the scoliosis kept developing radiologically even though the clinical improvement of the patient was considerable. In author's opinion, in such cases the clinical improvement is satisfactory and the time spent by children exercising was not wasted because a very good physical appearance of the child was obtained.

Applying FITS therapy appears advisable in children who were diagnosed with small angle of scoliosis as the results obtained in the group of children who were not using braces with the scoliosis angle of 10-25° were very good. It would probably significantly decrease the risk of further progression of scoliosis and the need for use of braces.

### Strong and weak points of the study

There are few published studies, which present the influence of the exercise alone on the improvement of the scoliosis angle. Presenting the treatment results of children within the range of 10-25°, who came in line with SRS criteria is a strong point of this study.

There are also weak points of this study:

First, the observation period is limited to a mean follow-up of 2.08 years. Many of these children did not obtain the skeletal maturity so they should be subjected to further observation. The FITS method has been in use since 2005. This is why it was difficult to compose big examination groups, which at the same time come in line with SRS criteria, had already accomplished the treatment period and were past the 2-3 year long observation period after finished treatment.

The second weak point of this study is the small number of subjects in B1 group-single curve scoliosis with Cobb angle of 26°-40°.

Next weak point-taking into account the group A-scoliosis with Cobb angle of 10-25° to provide post-treatment 3.8% progression > 5° in group A1 and 19.2% progression in group A2. We must consider whether progression after treatment, amounting to 3.8% in group A1 and 19.2% in group A2 is important for a child or parent. Considering the smallest initial Cobb angle in this group (10°), progression after 2 years therapy FITS > 5°, Cobb angle increased to 16-20°. Progression is at such an angle of curvature is not important, but if the observation period was longer-about 5 years, could prove that this progression is statistically significant (everything will depend on how old the child is and what is its degree of maturity of the bone).

Another weak point of the study is the lack of objective evaluation of the regularity and precision in performing the ordered exercises. Some of the children exercise with their parents while others work with therapists trained in the field of FITS concept. One cannot be sure to what extent the children or their parents were compliant in terms of performing the exercises or to what extent the therapists fulfilled recommendations.

## Conclusions

1. Preliminary results suggest that FITS could be an effective treatment, capable to alter the natural history of mild idiopathic scoliosis. Further studies are necessary showing results at maturity and beyond maturity.

2. FITS therapy improved the external morphology (esthetics) of the patients.

3. Radiological progression was more common in double scoliosis than in single curves.

## Competing interests

The author performs FITS therapy in her practice (office).

## Authors' contributions

MB: study design, data collection, data analysis and interpretation, manuscript drafting.
